# Integrated primary oral health services in South Africa: The role of the PHC nurse in providing oral health examination and education

**DOI:** 10.4102/phcfm.v5i1.413

**Published:** 2013-03-11

**Authors:** Lawrence K. Thema, Shenuka Singh

**Affiliations:** 1 School of Health Sciences, University of KwaZulu-Natal, South Africa

## Introduction

The occurrence, distribution and impact of oral diseases in communities qualify the need for collaboration of oral health workers with other primary health care service providers in the provision of health services. Integrated oral health planning and service delivery have the potential to improve access to oral health services and redress the historical inequities in oral health care.^1^ Oral disease levels in South Africa are considered to be of low severity when compared to other countries, however the prevalence and distribution of dental caries in particular are a huge public health concern.^1^ The National Oral Health Children’s Survey indicates an imbalance in the prevalence of dental caries within districts and across the various provinces.^2^ Given the historical inequities in oral health service delivery, a huge burden is placed on the public health system to deliver adequate and appropriate oral health services.

### Background

In developing countries where delivery of health services relies on the existence of a well-functioning workforce of nurses and an efficient pharmaceutical distribution system, it makes sense to integrate services with the well-functioning workforce, such as nursing services.^3^ The World Health Organization (2008) defines integrated services as ‘the organization and management of health services so that people get the care they need, when they need it, in ways that are user-friendly, achieve the desired results and provide value for money’.^3^ Integration of services does not necessarily mean that all different services have to be integrated in one package or delivered at the same place. However, it does mean that services have to be provided in a manner that is not disjointed and is easy to navigate by the user.^3^

### Scope

Given the historical imbalances in oral health care, the shortages of skilled workforce and the high unmet oral health needs, this paper provides an overview of the value of considering an alternate method of oral health service delivery. The paper focuses primarily on examining the potential role of nurses in primary health care settings to deliver essential oral health services, such as oral examinations and education. The value of integrated oral health care is also presented, where oral health care is an essential and integral part of general health care.

#### Impact of oral health on quality of life

Oral diseases are the most commonly occurring chronic diseases, affecting individuals and society with a resultant impact on general health and well-being.^4^ Oral diseases impact on society through compromised functioning of the oral cavity. People that have lost their teeth tend to avoid food that requires mastication, and this avoidance leads to inadequate nutritional intake. The resultant impact is poor general health.^4,5^

There is also a burden of indirect losses due to the loss of productivity during the time seeking oral health services, or unpaid salaries due to absence from duty.^4,5,6^ The loss of hours at school and time at work is mainly due to the clinical management of oral diseases being available during school and work hours.^4^ The impact is higher in communities with unmet oral health needs.^4^

Apart from the link between socio-economic status and unmet oral health needs, the lack of oral health professionals and facilities can also contribute to unmet oral health needs.^7,8^ Inadequate employment opportunities for oral health personnel in the public sector, amongst other challenges, has resulted in persistent staff shortages.^1^ A further fact is that oral health services remain a low priority in terms of budgetary allocations.^9^ Oral health workers therefore face challenges in service delivery that are further complicated by limited access to communities because of poor infrastructure.

#### The South African experience in oral health needs 

The National Children Oral Health Survey (1999–2002) indicated that children living in urban areas have slightly higher rates of dental caries than children living in rural areas and that the oral health needs vary widely from province to province. The greatest need was recorded in the Western Cape, where almost 80% of children needed oral health care, and the lowest need for dental care was recorded in Limpopo province.^2^ These survey findings have important implications for oral health planning in South Africa in terms of financing and human resource allocation requirement.^2^

High levels of oral diseases result in a greater demand for oral health professionals, equipment, oral health facilities and the financial resources to cater for the above needs. The value of human resource allocation for the delivery of oral health services cannot be understated. The current number of oral health professionals in South Africa is not adequate to meet the population’s oral health needs in the public sector (Table 1). The records further indicate that provinces such as Limpopo and Northern Cape have few, if any, oral hygienists employed in the public sector.^10^ This is of particular concern because preventive and/or promotive community oral health services are driven primarily by oral hygienists. This implies that preventive services are virtually absent in these provinces, thereby further justifying the need to explore other opportunities to ensure that basic oral health needs are met.

**TABLE 1:**
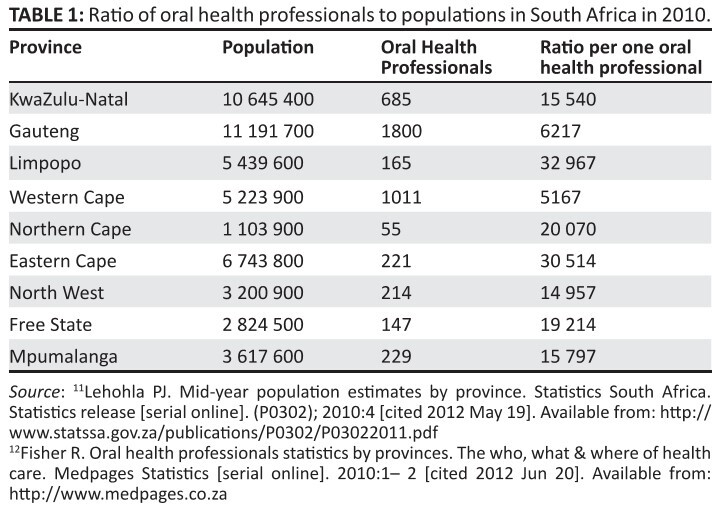
Ratio of oral health professionals to populations in South Africa in 2010.

The ratio of oral health personnel to the general population in South Africa is compared (Table 1).^11,12^ The ratio of oral health professionals to the population in the Northern Cape is of particular concern. These records indicate dire shortages of oral health personnel and thereby reiterating the need to consider integrated oral health service delivery. The ratio of oral health professionals with professional nurses to the general population on a provincial basis is compared (Table 2). The records indicate that a significant higher number of professional nurses are employed by the public sector in comparison to oral health professionals.

**TABLE 2:**
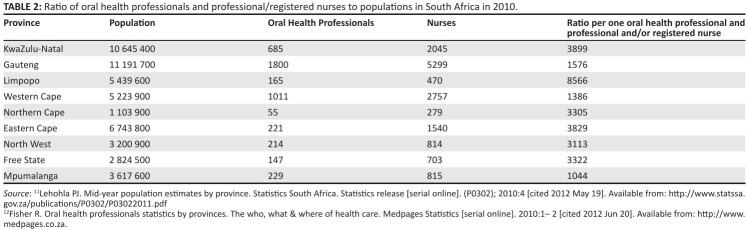
Ratio of oral health professionals and professional/registered nurses to populations in South Africa in 2010.

These records, in general, highlight the importance of identifying the human resource disparities occurring on a provincial basis. A possible reason for this disparity in human resource allocation may be rooted in the historical inequities in oral health service delivery. Repeated attempts were made to address these historical imbalances in oral health service delivery, but disparities still exist in post-apartheid South Africa.^1^ The urban-based, curative-driven, individual-focused delivery of oral health care suggest that oral health services in South Africa are still based on the principles of the bio-medical model.^1^ This is contrary to national policy statements on oral health care. 

The impact on the quality of life, exacerbated by the shortage of adequately trained oral health professionals, demands a re-look at oral health planning efforts in South Africa. There is a need for effective preventive interventions, early identification of oral diseases and appropriate referral for treatment.^10,13,14^ Collaboration and integration of oral examination and oral health education into primary nursing care can impact the human resource shortage in oral health services positively.^6,14^

#### Integrated health care

The common risk factor approach postulates that multiple disease presentations occur as a result of lifestyle practices. Thus oral diseases, especially dental caries, can co-exist with other chronic diseases such as obesity, diabetes and respiratory infections. Studies have also shown a distinct inter-relationship between severe periodontal disease and diabetes, as oral diseases comorbid with other general health conditions.^5,6^ It is therefore important that all health care providers consider the inclusion of oral examinations as part of the whole body examination.^5,6^

Health service integration is the bringing together of different health activities that share common health goals.^15^ Programmatic health integration involves the combination of different health programmes that share common goals. Health integration could also focus on administrative integration or policy integration. This paper focuses only on programmatic integration.

However, the process of integration faces a number of challenges in South Africa. Singh (2005) outlined various factors that have impeded oral health integration at district levels in South Africa. These include high workload resulting in low staff morale, insufficient information or skills, lack of administrative support to guide the integration process and a mismatch between the policy development and implementation processes.^15^

Integrated oral health service delivery is widely cited in health policy documents in South Africa, but research shows that the health policy process offers very little, if any, direction on the actual translation of these policy statements into implementable programmes.^1^ The implication of this lack of support and guidance by health management at district level is that the actual process of programmatic integration is left with the ‘grass-root’ health worker.^1^

#### Role of the primary health care nurse

Health care providers at primary health care centres are generally the ‘first line’ of health workers to meet basic health needs.^6,13^ Community members visit primary health care providers far more frequently than they would visit oral health professionals.^8^ These visits present opportunities for early identification of oral diseases, oral health education and referral for appropriate management of oral diseases.^4,7^

Nurses form a central component of primary health care centre services. However, many categories of nurses exist in primary health care settings. The scope of practice of these various categories of nurses is specific to the level of training. The following categories of nurses that could work in PHC clinics are:

- Registered general, midwifery psychiatry and community health nurses. They obtained the qualification as part of a basic comprehensive education in terms of the South African Nursing Council (SANC) regulation R425.00 which could be a degree or diploma.
- Or those that obtained a post basic diploma in community nursing science in terms of the SANC regulation R276.00. 
- Or those that did a course in clinical nursing science leading to registration of an additional qualification in community nursing science in terms of the SANC regulation R212.00.

These categories of nurses are collectively referred to as community nurses.^16^ The term ‘professional nurse’ is also used to address a registered nurse in South Africa. 

The public refers to the other category (those that obtained a diploma in clinical nursing science health assessment, treatment and care in terms of SANC regulation R48.00) as primary health nurses. They are also entitled to make diagnosis and prescribe treatment in specified circumstances (working in clinics where medical practitioners are not readily available).^16^

The registered nurse provides direct patient care that includes: patient examination, recording of symptoms and the provision of treatment and medication relevant to the scope of practice. Health education (both on an individual and group level) is an integral component of the registered nurse’s responsibilities. The registered nurse is assisted by enrolled nurses (staff nurses) and enrolled nursing assistants.^10^

Registered nurses’ direct contact with communities makes them the ideal category of staff to consider for integrated oral health service delivery. Hecksher et al. (2007) postulates that with adequate training in performing oral examinations, registered nurses have demonstrated a 95% success rate in disease identification and referrals.^17^ Early oral examinations will help early detection of oral disease, thereby preventing or minimising the development of serious oral health conditions that could require advanced clinical management or hospitalisation.^4,5^

Apart from early detection of oral diseases, the potential exists to implement comprehensive integrated preventive and promotive programmes thereby contributing to a reduction in the burden of oral diseases.^3,4^ The need to provide comprehensive health services with a strong preventive focus is entrenched in health policy statements in South Africa.^1^ The integration of specialised health services into primary health care is seen as an effort to reduce the isolation of services and increase accessibility to the communities, with the rationale being that such integration would be beneficial to both the community and oral health professionals.^5^

The value of integrating oral health education and oral examinations into nursing care at primary health care level is evident. However, programmatic health integration remains a philosophical concept that struggles to translate into practice in South Africa.^15^ The integration of specific oral health care into nursing care at primary health care level has the potential to address the current shortages in oral health human resources, but more importantly - this approach provides a viable platform to ensure comprehensive management of the patient.^18^

This paper highlights the need for integrated primary oral health care, but does not focus on the perceptions, attitudes and beliefs of registered nurses towards health integration. Further research is required to facilitate the integration of specific primary oral health care into facility-based and/or clinic-based primary health care delivery. The potential opportunities and barriers should be explored:

- The interrelationship between oral health and general health, and the impact of chronic lifestyle-induced diseases should be an integral component of all health awareness activities.
- The role of registered nurses in integrated primary oral health care delivery necessitates the need for policy discussion at all levels of the health system. 

## Conclusion

Inputs in the education curriculum and continuing education for registered nurses on issues such as oral examination and oral health education, can contribute to turning programmatic health integration into a reality. 
